# Resource-dependent heterosynaptic spike-timing-dependent plasticity in recurrent networks with and without synaptic degeneration

**DOI:** 10.3389/fncom.2025.1593837

**Published:** 2025-07-22

**Authors:** James Humble

**Affiliations:** Independent Researcher, Randolph, MA, United States

**Keywords:** spike-timing-dependent plasticity, homeostasis, heterosynaptic, recurrent network, learning, spiking, synaptic degeneration, neurodegeneration

## Abstract

Many computational models that incorporate spike-timing-dependent plasticity (STDP) have shown the ability to learn from stimuli, supporting theories that STDP is a sufficient basis for learning and memory. However, to prevent runaway activity and potentiation, particularly within recurrent networks, additional global mechanisms are commonly necessary. A STDP-based learning rule, which involves local resource-dependent potentiation and heterosynaptic depression, is shown to enable stable learning in recurrent spiking networks. A balance between potentiation and depression facilitates synaptic homeostasis, and learned synaptic characteristics align with experimental observations. Furthermore, this resource-based STDP learning rule demonstrates an innate compensatory mechanism for synaptic degeneration.

## 1 Introduction

The concept of spike-timing-dependent plasticity (STDP) has been thoroughly researched and frequently serves as a foundation for learning in computational models. Various studies adopt STDP in diverse formats. For instance, it may be utilized with either an additive or a multiplicative rule for updates: Potentiation or depression may depend on or be independent of a synapse's weight. Different STDP implementations can lead to varied outcomes, with some rules more closely reflecting phenomena observed in experiments. These variations include (1) synaptic weight distributions, (2) the presence of non-potentiable synapses, (3) silent synapses, (4) synaptic persistence, and (5) competition between synapses:

1. Empirically identified synaptic weight distributions generally display a uni-modal pattern that peaks close to zero, characterized by numerous weak synapses and a few strong connections forming a long tail (Buzsáki, 2004; Yasumatsu et al., 2008; Kasai, 2023). In computational models, synaptic distributions change based on whether STDP is implemented additively or multiplicatively. In feedforward networks, additive STDP often produces bi-modal distributions, with peaks located near zero and the upper limit of synaptic weight (Rossum et al., 2000; Barbour et al., 2007; Morrison et al., 2008). In contrast, multiplicative STDP often generates uni-modal distributions with a peak situated between zero and the upper bound (Rossum et al., 2000; Barbour et al., 2007; Morrison et al., 2008). Gütig et al. (2003) used a non-linear STDP model to interpolate between these two extremes and found that weight distributions transition between bimodal and unimodal, albeit not similar to the characteristic experimentally observed long tailed distribution. In recurrent networks, additive STDP can produce a uni-modal distribution with a peak at the upper-bound and multiplicative, uni- or multi-modal distributions (Morrison et al., 2007).
2. Silent synapses are primarily defined by their absence of α-amino-3-hydroxy-5-methyl-4-isoxazolepropionic acid (AMPA) receptors, as comprehensively discussed by Montgomery and Madison (2004). Despite the scarcity of AMPA receptors, these synapses often retain a degree of plasticity due to the presence of *N*-methyl-D-aspartate (NMDA) receptors (Kim et al., 2025). In a study by Brunel et al. (2004), a prominent and sharply delineated subset of silent synapses was integrated into an empirically derived distribution by assessing those potentially undetected because of technological limitations and their deficiency in AMPA receptors. Such a peak is observed in spine volume (Yasumatsu et al., 2008) and synaptic efficacy (Barbour et al., 2007).
3. Research has indicated that certain synapses may not be capable of potentiation. For instance, Debanne et al. (1999) reported the inability to induce potentiation in 24 % of the synapses examined. Debanne et al. propose that this attributed to synapses individually reaching saturation. Conversely, computational models often employ either a universal upper limit across all synapses or a global normalizing mechanism and neglecting these constraints may lead to excessive activity and potentiation (Rossum et al., 2000).
4. The persistence of synapses is typically considered to be fundamentally important for memory. In their study, Billings and Rossum (2009) investigated the durability of synaptic weights governed by STDP principles and found that additive STDP facilitates stability, whereas multiplicative STDP causes instability due to rapid weight variations. Gütig et al. (2003) found that a non-linear STDP implementation permitted stable learning when in the multiplicative regime. Empirical evidence from long-term potentiation (LTP) studies indicates a two-phase persistence: an initial phase that diminishes swiftly and seems to be reliant on neuronal activity (Dong et al., 2015), and a later phase capable of sustaining LTP over prolonged periods, possibly extending to a year (Abraham et al., 2002). There is, however, strong evidence demonstrating that spine volumes fluctuate in the absence of activity and plasticity [reviewed by Kasai (2023)] and that such fluctuations combined with STDP can be stable (Humble et al., 2019). In this case, the mean spine volume of a group of neurons must be persistent rather than the individual synaptic strengths.
5. Whereas STDP following an additive rule is known for its strong competitive interactions, STDP governed by a multiplicative rule typically exhibits limited competition [although see Gütig et al. (2003) for competitive non-linear STDP], prompting the incorporation of supplementary mechanisms such as synaptic scaling (Turrigiano, 2008) or intrinsic fluctuations to achieve the competition essential for learning (Rossum et al., 2000; Humble et al., 2019). The inherently competitive aspect of additive STDP typically necessitates enforcing a stringent upper limit on synaptic strength to control excessive potentiation. In contrast, multiplicative STDP operates under more flexible upper limits determined by weight dependency. A drawback of deploying global limits is their assignment of preset values before the learning process, which may not align with biological realism. Moreover, as mentioned above, Debanne et al. (1999) demonstrated the possibility of individual synaptic upper limits.

Furthermore, to control runaway activity, mechanisms such as synaptic scaling (Turrigiano, 2008), inhibition (Bannon et al., 2020; Eckmann et al., 2024), or the Beinenstock-Cooper-Munro rule (Cooper and Bear, 2012) are typically included with Hebbian-based learning rules in recurrent networks. However, these can operate on a slower timescale than that required for STDP (Zenke et al., 2013). In addition, weight normalization is often included in computational models of plasticity in recurrent models; however, because it requires a continuous global sum of weights across a neuron, it is less biologically plausible than a local mechanism.

The above picture is further complicated by experiments showing that heterosynaptic plasticity can occur in unstimulated spines near a stimulated one [reviewed by Chater and Goda (2021)]. Essentially, given some homosynaptic activity in a subset of spines, heterosynaptic changes have been observed in unstimulated ones. The distance dependence and direction of these heterosynaptic changes are potentially competitive (Chater et al., 2024).

Motivated by these experimental results, this paper explores ongoing research into computational disparities by utilizing a learning methodology that integrates “resources” alongside heterosynaptic plasticity in a spiking recurrent network. In a computational model of individual neurons (Chen et al., 2013) and a feedforward network [Chapter 5 of Humble (2013)], it has previously been shown that heterosynaptic plasticity can be beneficial in controlling activity and plasticity.

In addition to networks with static connectivity, progressive loss of synapses is a hallmark of many neurodegenerative diseases, including Huntington's, Parkinson's, and Alzheimer's (Herms and Dorostkar, 2015; Meftah and Gan, 2023), and neuropsychiatric disorders such as schizophrenia and depression (Penzes et al., 2011). To counteract synaptic loss, the existence of compensatory mechanisms has been suggested that include enlargement of the remaining spines and increased spinogenesis (Bhembre et al., 2023).

By advancing STDP through the local incorporation of limited resources for potentiation and merging it with heterosynaptic depression that affects neighboring synapses, the results reveal that this model effectively resolves the discrepancies mentioned above while presenting an innate local mechanism for both synaptic homeostasis and synaptic degeneration compensation.

## 2 Methods

**Figure 1:**
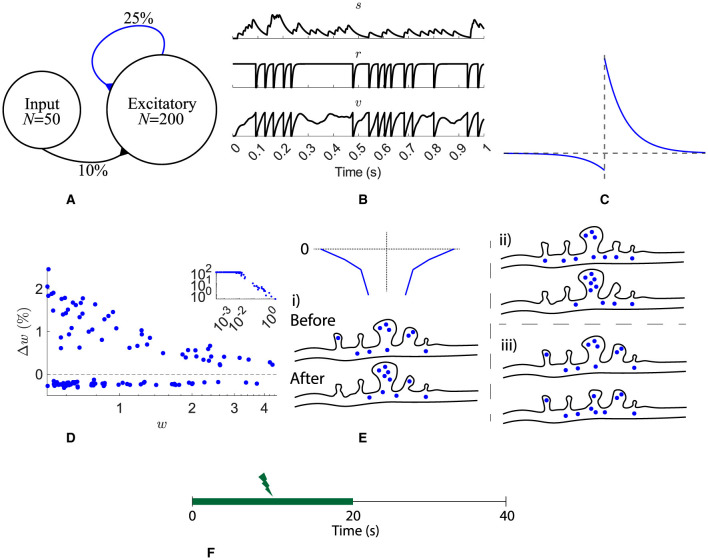
Network, neuron, and synaptic models and stimulation protocol. **(A)** A pool of 200 excitatory neurons receive input from 50 independent Poisson processes. All recurrent synapses undergo STDP. **(B)** A synaptic input, *s*, and one neuron's refractoriness, *r*, and membrane potential, *v*. **(C)** STDP learning window. **(D)** Synaptic change dependence on weight. **(E)** Spines on a dendrite are depicted either before or after a STDP learning event. The spine volume and number of resources is analogous to synaptic strength. Three different scenarios for heterosynaptic plasticity are shown: (i) potentiation accompanied by heterosynaptic depression of resources (blue circles) from neighbors (dependent on a decaying exponential as a function of distance, top left), (ii) potentiation with resources taken from the pool (resources in the dendrite), and (iii) depression with resources being relocated in to the dendrite pool. **(F)** Input is provided for the first 20 s.

The network structure (Figure 1A) consists of a pool of *N*_exc_ = 200 excitatory neurons recurrently connected with plastic excitatory synapses with a probability of 25 %. Independent Poisson spike trains are presented to the pool with an input connection probability of 10 % from *N*_in_ = 50 spike trains. All connections have axonal delays drawn from a uniform distribution from 1 ms to 5 ms (Lemaréchal et al., 2021).

Throughout the following *n* notates a neuron's index, *i* notates an input synapse's index, *j* notates a recurrent excitatory synapse's index, and *i*/*j* notates either *i* or *j*.

Excitatory neurons are refractory leaky integrate-and-fire cells (Equation 1). The membrane potential of each neuron, *v*, follows low-pass dynamics with a time constant τ_*v*_ = 25 ms (Rall, 1969) and is reset when it reaches the firing threshold, θ = 1 (Figure 1B bottom).


(1a) $$\begin{array}{l} \tau_{v}\frac{dv_{n}}{dt}=-v_{n}+r_{n}\left(\overset{N_{\text{in}}}{\underset{i}{\sum }}s_{i}+\overset{N_{\text{exc}}}{\underset{j}{\sum }}s_{j}\right) \end{array}$$

(1b) $$\begin{array}{l} v_{n}\to 0\;\text{if }v_{n}\geq \theta \end{array}$$

An absolute refractory period of 3 ms is followed by a relative refractoriness period, τ_*r*_ = 5 ms (Equation 2 and Figure 1B middle).


(2) $$\begin{array}{l} \tau_{r}\frac{dr_{n}}{dt}=1-r_{n} \end{array}$$

All input and recurrent afferents were modeled as synaptic currents, *s*′ and *s* (Equation 3 and Figure 1B top). τ_*sr*_ = 2.6 ms is the time constant for the synaptic current rise and τ_*sf*_ = 31.3 ms the decay constant (Hunt et al., 2022). The input of each synapse consists of binary presynaptic neuron spikes, *I*, scaled by the synapse's weight, *w*.


(3a) $$\begin{array}{l} \tau_{sr}\frac{ds_{i/j}^{\prime }}{dt}=-s_{i/j}^{\prime }+I_{i/j}\;w_{i/j} \end{array}$$

(3b) $$\begin{array}{l} \tau_{sf}\frac{ds_{i/j}}{dt}=-s_{i/j}+s_{i/j}^{\prime } \end{array}$$

Recurrent excitatory synapses are plastic. Pairs of presynaptic and postsynaptic spikes evoke changes in synaptic weight, Δ*w* = *f*(τ), given as a function of their temporal distance, τ = *post-spike time*−*pre-spike time* (Figure 1C). Equation 4 describes the STDP function used, where τ_*STDP*_ = 20 ms (Bi and Poo, 1998). Depression is weight dependent, as observed by Bi and Poo (1998) (Figure 1D). Recurrent weights were bounded [0, +∞). See below for a description of the inclusion of 0.18 in depression.


(4) $$f(\tau )=\{\begin{array}{ll} \text{exp}\left(\frac{-\tau }{\tau_{STDP}}\right) & \text{if }\tau \geq 0\\ 0.18\text{ exp}\left(\frac{\tau }{\tau_{STDP}}\right)\;w & \text{if }\tau <0 \end{array}$$

Previous models have included noisy STDP updates, for example, Rossum et al. (2000), to replicate the spread in weight dependence observed experimentally by Bi and Poo (1998), however, without these noisy updates, resource-dependent STDP replicates the weight dependence spread (Figure 1D).

The initial input weights are set with random values drawn from a uniform distribution between 0 and 1. All recurrent weights, *w*, are initially assigned 0 as akin to a newly formed network.

This typical STDP implementation is extended as in Humble (2013) to include the requirement of resources for potentiation paired with potentiation-driven heterosynaptic depression in neighboring synapses. Firstly, a pool of resources is included, *p* per neuron. There is a single pool of resources per neuron that is common across all synapses on a single neuron. Similarly to many receptors and proteins that undergo degradation or recycling, the pool's resources decay with a time constant of τ_*p*_ = 10 s (Equation 5).


(5) $$\begin{array}{l} \tau_{p}\frac{dp_{n}}{dt}=-p_{n} \end{array}$$

The amounts of the initial resource pool are assigned by Equation 6 where ξ is a random number from a standard normal distribution. The constant multiple was found through a parameter search to ensure that the weights were strong enough that the activity continued after stimulation stopped. A log normal distribution is chosen as skewed distributions are observed for many aspects of brain dynamics (Buzsáki and Mizuseki, 2014).


(6) $$\begin{array}{l} p_{n}=2\;e^{\xi } \end{array}$$

When updating the weights with resource-dependent STDP, there are three scenarios, Figure 1E where → denotes letting a variable take a new value:

- When potentiating a synapse, its neighbors are actively depressed by an exponential function of their distance to the potentiating synapse, and the potentiating amount to a maximum distance of 3 synapses (Equation 7).

(7) $$\text{if}\;\sum_{k=1}^{3}w_{j\pm k}\geq 0\{\begin{array}{l} \text{for}\;k\in \left\{1,2,3\right\}\;w_{j\pm k}\to w_{j\pm k}\\ -f(\tau )\;\left(e^{-\text{abs(}k\text{)}}/\sum_{k\prime =1}^{3}e^{k\prime }/2\right)\\ w_{j}\to w_{j}+f(\tau ) \end{array}$$

- When potentiating a synapse and its neighbors have no resources left, the potentiating synapse acquires resources from the pool, if available (Equation 8).

(8) $$\text{if}\;\sum_{k=1}^{3}w_{j\pm k}=0\{\begin{array}{l} \text{if}\;p_{n}\geq f(\tau )\{\begin{array}{l} w_{j}\to w_{j}+f(\tau )\\ p_{n}\to p_{n}-f(\tau ) \end{array}\\ \text{else}\{\begin{array}{l} w_{j}\to w_{j}+p_{n}\\ p_{n}\to 0 \end{array} \end{array}$$

- When a synapse is depressed, its resources are relocated to the pool for reuse (Equation 9).

(9a) $$\text{if}\;w_{j}\geq f(\tau )\{\begin{array}{l} p_{n}\to p_{n}+f(\tau )\\ w_{j}\to w_{j}-f(\tau ) \end{array}$$

(9b) $$\text{else}\;\{\begin{array}{l} p_{n}\to p_{n}+w_{j}\\ w_{j}\to 0 \end{array}$$

Combinations of the above scenarios are possible. For example, when potentiating, it is possible that neighboring spines provide only 50% of the resources required for potentiation; in this case, additional resources will be taken from the pool. Furthermore, if the pool and neighboring spines are depleted, potentiation is not possible. Moreover, when neighboring spines or the pool have insufficient resources but are non-zero, the amount of resources acquired are equal to those available, and the neighboring spines or pool are depleted. Finally, given a case where the neighboring spines' resources combined with any available from the pool are not sufficient for potentiation, the amount of potentiation is limited.

For simplicity, time constants for resource mobility are neglected; hence, resources can transfer between a synapse and the pool, and vice versa, within a single time step.

With local heterosynaptic plasticity, depression dominates because potentiation events are accompanied by depression, and thus depression has to be decreased. It has previously been found that 0.18 was a suitable multiple for depression such that potentiation and depression were balanced (Humble, 2013). This reduced depression (Figure 1C) matches the experimental observations of decreased depression relative to potentiation (Bi and Poo, 1998).

The rate of the input spike trains is 50 Hz for the first 20 s (Figure 1F).

All simulations used forward Euler integration with a time step of *dt* = 0.1 ms and were implemented in MATLAB.

## 3 Results

**Figure 2:**
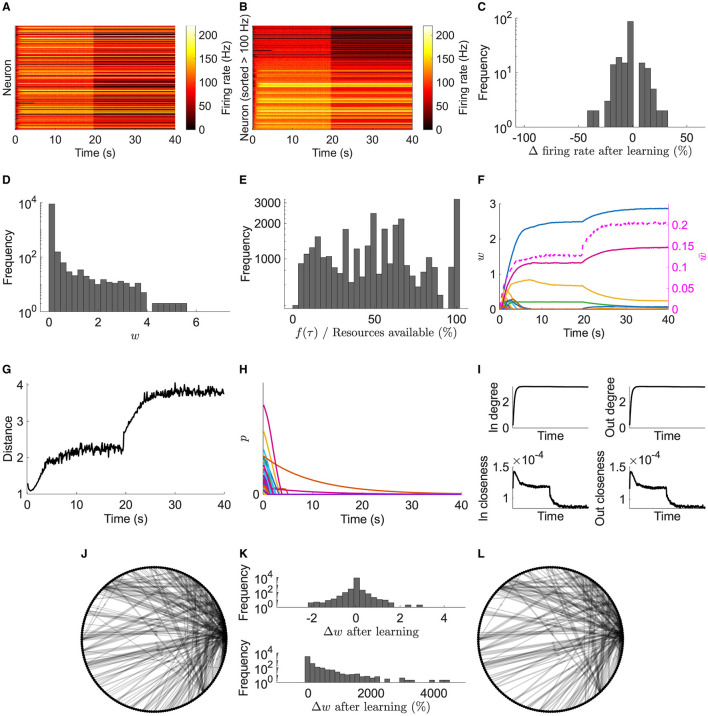
Typical network dynamics. **(A)** Firing rates of the network's neurons. **(B)** Sorted firing rate of the network's neurons for when they first fire ≥100 Hz. **(C)** Change in the firing rate from after learning to the end of the simulation. **(D)** Weights, *w*, at the end of the simulation. **(E)** Actual potentiation amount as a percentage of that determined by the STDP window. **(F)** Left axis: example of 100 synapses' weights. Right axis/dashed line: mean of nonfilopodia synapses' weights. **(G)** Distance between nonfilopodia spines. **(H)** Resource pools, *p*. **(I)** Mean in degree, out degree, in closeness, and out closeness during the simulation. **(J)** Network of neurons after learning. **(K)** Weight change after learning. **(L)** Network of neurons at the end of the simulation. **(J)** and **(L)** Only the strongest 10 % are shown. Line width represents weight.

During stimulation of a typical network, most neurons in the excitatory pool fire with increased firing rates (Figures 2A, B). After stimulation, a subset continues to fire representing a learned memory. Not all neurons are recruited into the memory, due to initial random input and recurrent connectivity and random activity-driven plasticity. Furthermore, after learning, the firing rates of many neurons change (Figure 2C); nevertheless, the memory is preserved.

**Figure 3:**
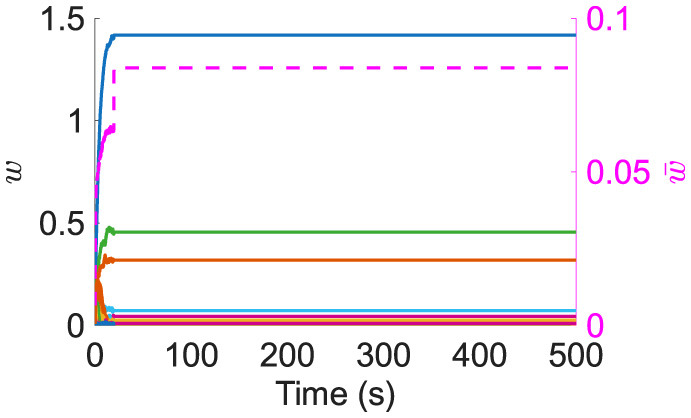
Left axis: example of 100 synapses' weights during a long simulation. Right axis/dashed line: mean of nonfilopodia synapses' weights.

After learning, weight statistics match those found experimentally—five observations were identified in the introduction:

1. The weight distribution for recurrent connections at the end of the simulation is unimodal (Figure 2D) matching those observed experimentally (see Supplementary Figure 1 for additive and multiplicative STDP results in this model without resource dependence and Supplementary Figure 2 for non-linear STDP with resource dependence).
2. The distribution also shows a large peak of empty synapses: silent synapses.
3. Resource-based STDP endows reduced and nonpotentiability (Figure 2E). Specifically, the actual potentiation amount as a percentage of that determined by the STDP window function demonstrates that sometimes not enough resources are available to fully potentiate a synapse; some times very little or no potentiation was observed.
4. Resource-based STDP is stable, with weights remaining persistent in a longer simulation (Figure 3).
5. Weights converge to individual upper bounds despite the positive feedback loop present due to plasticity. It was observed that weights reach their own saturation points (Figure 2F). Furthermore, due to the distance dependence of heterosynaptic depression, analysis indicates that resource-based heterosynaptic STDP promotes synaptic sparsity and synaptic competition for limited resources, with functional spines becoming spatially distributed along the dendrite. The average distance between a nonfilopodia spine (with a non-zero weight) and its closest nonfilopodia spine neighbor increases during learning (Figure 2G).

Most of the resources available in the neurons' pools quickly decrease due to plasticity driven changes (Figure 2H). Some pools may not be used up due to plasticity and instead naturally decay.

During the initial learning phase, the network's in/out degree increases and stabilizes ≈3, and the closeness (where the length of the paths is the axonal delay) decreases (Figure 2I). The decrease in closeness demonstrates that learning optimizes for short delays.

After the initial learning phase, the network is further optimized with an increase in nonfilopodia spine distance and a decrease in closeness. Furthermore, the network structure changes during this optimization phase with weights increasing or decreasing (Figures 2J–L). Most strong connections are stable with a few changing; the majority of optimization changes are with weaker synapses.

**Figure 4:**
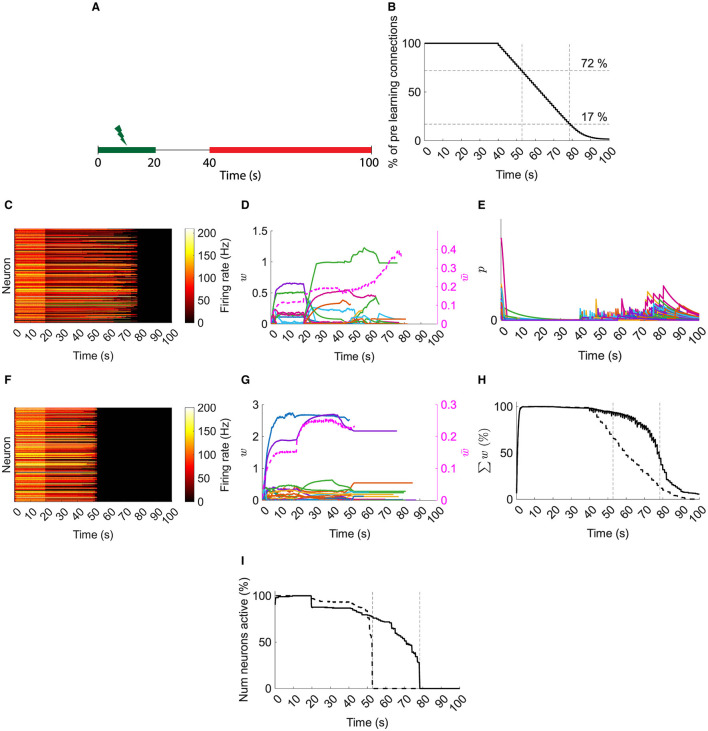
Typical network dynamics with and without resource pool replenishment while removing connections. **(A)** Input is provided for the first 20 s. Synapses are progressively deleted from 40 s on (red). **(B)** Progressive synaptic loss. **(C)** Firing rates of the with replenishment of the resource pool. **(D)** Left axis: example of 100 synapses' weights with resource pool replenishment. Right axis/dashed line: mean of nonfilopodia synapses' weights. **(E)** Resource pools, *p*, with replenishment of the resource pool. **(F)** Firing rates of the network's neurons without replenishment of the resource pool. **(G)** Left axis: example of 100 synapses' weights without replenishment of the resource pool. Right axis/dashed line: mean of nonfilopodia synapses' weights. **(H)** Sum of weights as a percentage of the maximum sum of weights. Solid line, with resource replenishment; dashed line, without resource replenishment. **(I)** The number of active neurons as a percentage of the maximum number of active neurons. Solid line: with resource replenishment; Dashed line: without resource replenishment. **(B)**, **(H)**, and **(I)** Vertical lines are when all neurons stopped firing either with or without resource replenishment. **(D)** and **(G)** Weights are shown while they exist and the mean of nonfilopodia synapses' weights is shown while neurons are active.

These results in a typical network demonstrate that resource-based STDP is capable of stable learning in recurrent networks with an innate homeostasis mechanism controlling runaway activity and potentiation. Next, to model spine loss in neurodegenerative diseases, synapses are progressively removed (Figures 4A, B).

In a typical network with resource-based heterosynaptic STDP, the memory is maintained until ≈17 % of the original connections remain (Figure 4C). As synapses are removed, their resources replenish the pool and allow further compensatory potentiation with an increase in mean synaptic weight and transient increases in the pool of resources (Figures 4D, E). However, when this replenishment is blocked, the memory is only maintained until ≈72 % synapses remain (Figure 4F)—with no increase in the remaining synaptic weights (Figure 4G).

This is further illustrated when comparing the sum of weights as a percentage of the maximum sum (Figure 4H). Under the replenishment-blocking condition, the total synaptic input disappears ≈4 times faster than under the control condition. Moreover, loss of neuron activity is sudden without replenishment of resources; whereas with replenishment of resources there is a progressive loss (Figure 4I).

## 4 Discussion

Discrepancies were reconciled between computational STDP models and empirical observations by using a STDP framework based on resource-dependent heterosynaptic STDP in a recurrent spiking network. By integrating resource-dependent potentiation with heterosynaptic depression at nearby synapses, neurons performed a learning task while preserving synaptic weight attributes and statistical traits consistent with biological data.

Resource-dependent heterosynaptic STDP results in weight distributions characterized by a singular peak and a pronounced tail. The distribution also shows a large peak at zero of spines empty of resources (silent synapses) similar to those estimated by Brunel et al. (2004) and similar to an abundance of filopodia as seen by Yasumatsu et al. (2008) and reviewed by Kasai (2023).

Furthermore, this approach to heterosynaptic STDP incorporates robust competitive dynamics and synaptic homeostasis, leading to varying intrinsic upper limits for individual synapses at which a synapse is no longer potentiable. In addition, the STDP rule promotes sparse spatial encoding in afferents and therefore strong competition between neighboring spines for limited resources. This homeostasis among weights is independent of any additional normalization mechanism or universal constraints to regulate learning. Such synaptic homeostasis is a compelling mechanism for stabilizing neural activity and plasticity within recurrent networks.

Finally, given progressive synaptic loss, resource-based STDP demonstrated an innate compensatory mechanism due to replenishment of resources; this mechanism has been hypothesized to counteract a loss in input (Bhembre et al., 2023). Loss of synaptic input is associated with early stages of neurodegenerative diseases, for example, Alzheimer's disease (Spires et al., 2005). Resource-based STDP demonstrated the ability to maintain total synaptic input by replenishing resources in the pool after synaptic removal. Moreover, resource-based STDP demonstrated a sufficient substrate for observations showing enlargement of synapses following insults such as deafferentation and sensory deprivation (Chen and Hillman, 1982; Barnes et al., 2017) and the hypothesized spine enlargement in Alzheimer's disease (Bhembre et al., 2023). Finally, resource-based STDP exhibited a progressive loss in, rather than a sudden loss in, neuron activity. How this relates to aberrant activity observed in Alzheimer's disease (Korzhova et al., 2021) is unknown, and exploring this offers an interesting future extension to this work.

Given that the proposed STDP rule aligns with experimentally observed weight statistics and supports a hypothesized neurodegenerative compensatory mechanism, its potential biological plausibility warrants examination. Royer and Paré (2003) observed in amygdala slices that the induction of long-term depression (potentiation) resulted in corresponding long-term potentiation (depression) at distally located dendritic sites, determined by the distance from the initial induction site. In contrast, Hou et al. (2008) reported no such synaptic changes at adjacent sites in cultured hippocampal neurons derived from rat embryos.

The research carried out by Oh et al. (2015) in P6-P7 hippocampal slice cultures aligns with the proposed STDP model. Stimulation-induced potentiation in specific spines was demonstrated to cause a reduction in the size of adjacent unstimulated spines. This observation can be explained by two potential mechanisms: first, the competition among nearby spines for a scarce resource, or second, an activity-triggered signal facilitating the reduction of adjacent spines. The findings of Oh et al. lend credence to the second hypothesis. They determined that blocking calcineurin, IP_3_ receptors or group I metabotropic glutamate receptors hinders heterosynaptic shrinkage, without affecting the potentiation process when Ca^2+^/calmodulin-dependent protein kinase II (CaMKII) is inhibited. This evidence indicates that synaptic potentiation and the associated decrease in nearby synaptic strength operate through distinct pathways.

Bian et al. (2015) observed that competition among spines for cadherin/catenin complexes plays a crucial role in orchestrating both the maturation of individual spines and the pruning of adjacent spines. Their *in vivo* studies revealed that variations in cadherin/catenin complex concentrations between neighboring spines lead to a redistribution of β-catenin, which in turn influences whether a spine matures or is trimmed. Crucially, they demonstrated that this process depends on neuronal activity and the distance between the enlarging spine and the nearby one that is slated to be eliminated. Furthermore, this competitive mechanism was not limited to individual neurons; it occurred in neighboring neurons receiving similar axonal input.

Within the framework of long-term potentiation (LTP), the relocation of AMPA receptors to synaptic sites is associated with increased synaptic activity (Hayashi et al., 2000; Sutton and Schuman, 2006; Shi et al., 2001). In this process, CaMKII experiences autophosphorylation when intracellular Ca^2+^ levels rise through NMDA receptor-mediated channels, culminating in the phosphorylation of GluR1 (Roche et al., 1996). AMPA receptors can originate from multiple sources, such as recycling endosomes (Park et al., 2004) and the trans-Golgi network (Horton and Ehlers, 2004). Our model consolidates these different origins into a common pool, taking advantage of evidence that AMPA receptors can traverse long distances, facilitated by movement along dendritic membranes (Choquet and Triller, 2003) and microtubule pathways (Washbourne et al., 2002). Regarding the mechanism that underlies heterosynaptic depression, Oh et al. (2015) observed that blocking calcineurin, IP_3_Rs, or group I metabotropic glutamate receptors hindered the contraction of adjacent spines. Additionally, Bian et al. (2015) propose the existence of a single molecular structure that fulfills dual roles, both as a “resource” and as a transmitter of depressive signals. Finally, recent experimental and computational evidence suggests that Ca^2+^ activity is a key component of competitive heterosynaptic plasticity (Chater et al., 2024).

In addition to resource-dependent heterosynaptic STDP, neuromodulation may apply additional constraints on the model. Brzosko et al. (2019) suggest that neuromodulation may bridge the millisecond STDP time scales and slower behavioral learning and may have effects before, during, and after STDP-induced changes. Before: plasticity priming via a- and B-adrengergic receptors (Seol et al., 2007) may move resources closer (for LTP) to or away (for long-term depression) from primed spines. During: nicotine increases the threshold for STDP induction (Couey et al., 2007) and noradrenaline widens the LTP time window (Liu et al., 2017), both of which can be additive to the proposed STDP rule. After: activation of dopamine receptors after an STDP event has been shown to convert STDP-dependent depression to potentiation (Brzosko et al., 2015)—including dopamine would be an interesting extension to the model, especially as it has been proposed as a synaptic tagging mechanism that can solve the distal reward problem (Izhikevich, 2007). See Brzosko et al. (2019) for a review of STDP neuromodulation. Furthermore, the reduction in cholinergic tone in Alzheimer's disease may contribute to STDP changes that lead to spine loss due to the age-dependent loss of STDP potentiation (Buskila et al., 2013).

The depiction of STDP herein does not include explicit modeling of biological signaling pathways or diffusible molecules that might cause depression in neighboring synapses after activity-driven potentiation, nor does it specify a precise timeline for such processes. Furthermore, it does not incorporate the transfer of resources into or out of a dendritic spine. Introducing these complexities could impose interesting limitations on the model, suggesting areas for future investigation.

Although a precise “resource” is not identified, nor is a specific depression signal characterized, multiple hypotheses suitable for experimental examination can be proposed under healthy and synaptic degenerative conditions.

- As described by Oh et al. (2015), suppressing the heterosynaptic depression signal could allow learning driven by potentiation under healthy conditions to continue until resources in the pool(s) are exhausted. Subsequent learning would require the creation of new resources. As a result, while the pace of learning may reduce under signal-suppressed conditions compared to when active signals are present, it would not come to a complete stop until all resources are used with no change in neighboring spines.
- Suppression of the depression signal under synaptic degenerative conditions might interfere with memory retention during synaptic loss if the signal is required for resource replenishment.
- Excessive activation of the depression signal in both healthy and synaptic degenerative conditions could result in a temporary surplus of resources that would persist until they are used by potentiating synapses or degrade over time.
- A reduction in total resources could hinder learning under healthy conditions, although it would not stop if vital resources are actively liberated through heterosynaptic depression.
- A reduction in total resources under synaptic degenerative conditions could accelerate the total loss of synaptic input and therefore the loss of neuronal/memory activity.
- In contrast, an increase in available resources might lead to uncontrolled neuronal activity and potentiation under health conditions.
- Under conditions of synaptic degeneration, an increase in available resources can contribute to memory retention.

## Data Availability

The code and datasets generated for this study can be found in https://github.com/JamesHumble/Humble-resource-based-STDP.
