# Supplementary material for: Resource-dependent heterosynaptic spike-timing-dependent plasticity in recurrent networks with and without synaptic degeneration
Source: Front Comput Neurosci. 2025 Jul 22;19:1593837. doi: 10.3389/fncom.2025.1593837 (PMC12321831; doi:10.3389/fncom.2025.1593837)
Supplement: Supplementary file 1 [file Data_Sheet_1.pdf]

Supplementary Material

1 METHODS

1.1 Additive STDP

Equation S1 describes the STDP function used. The learning rate $A = 0.2$ was assigned so that the learning was slow. Recurrent weights were bounded $[0, 1]$.

$$f(\tau) = \begin{cases} A \exp\left(\frac{-\tau}{\tau_{STDP}}\right) & \text{if } \tau \geq 0 \\ A \exp\left(\frac{\tau}{\tau_{STDP}}\right) & \text{if } \tau < 0 \end{cases} \tag{S1}$$

1.2 Multiplicative STDP

Equations S2 and S3 describe the STDP function used where w is the weight that is being updated. The learning rate $A = 0.05$ was assigned so that the learning was slow. Recurrent weights were bounded $[0, 1]$.

$$f(\tau) = \begin{cases} A \exp\left(\frac{-\tau}{\tau_{STDP}}\right) & \text{if } \tau \geq 0 \\ A \exp\left(\frac{\tau}{\tau_{STDP}}\right) \; w & \text{if } \tau < 0 \end{cases} \tag{S2}$$

$$f(\tau) = \begin{cases} A \exp\left(\frac{-\tau}{\tau_{STDP}}\right) & \text{if } \tau \geq 0 \\ A \exp\left(\frac{\tau}{\tau_{STDP}}\right) \; \frac{w}{0.5} & \text{if } \tau < 0 \end{cases} \tag{S3}$$

1.3 Non-linear STDP

Equation S4 describes the STDP function used similarly to Gütig et al. (2003) where w is the weight that is being updated. The learning rate $A = 0.2$ was assigned so that the learning was slow. Recurrent weights were bounded $[0, +\infty)$. Because weights are unbounded and it was found that weights never potentiate to 20, 20 was chosen as a limit to the potentiation multiplicative effect allowing a comparative implementation.

$$f(\tau) = \begin{cases} A \exp\left(\frac{-\tau}{\tau_{STDP}}\right) \; (20 - w)^{\mu} & \text{if } \tau \geq 0 \\ A \exp\left(\frac{\tau}{\tau_{STDP}}\right) \; w^{\mu} & \text{if } \tau < 0 \end{cases} \tag{S4}$$

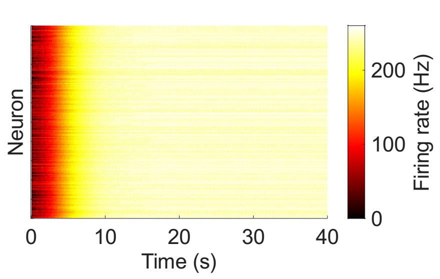

Figure 1a.

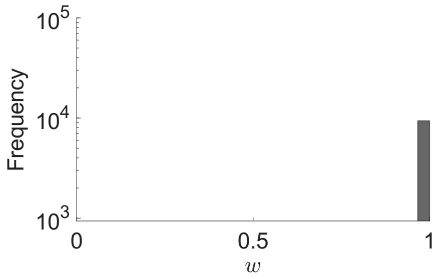

Figure 1b.

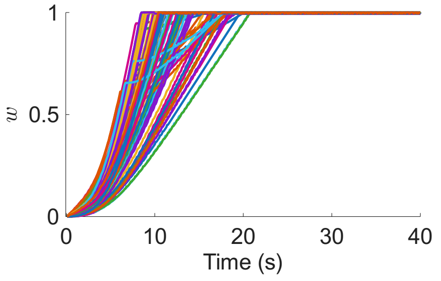

Figure 1c.

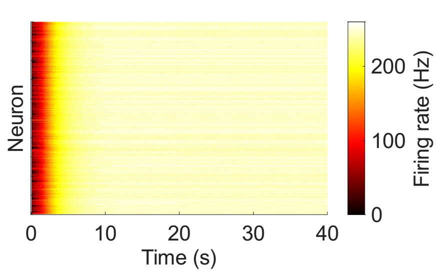

Figure 1d.

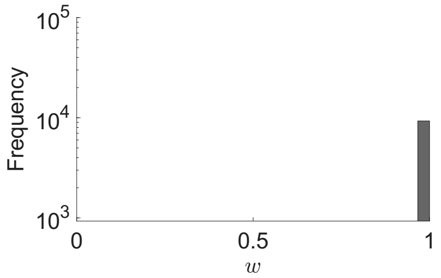

Figure 1e.

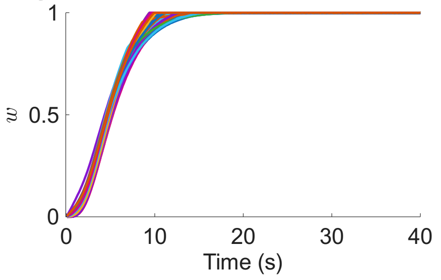

Figure 1f.

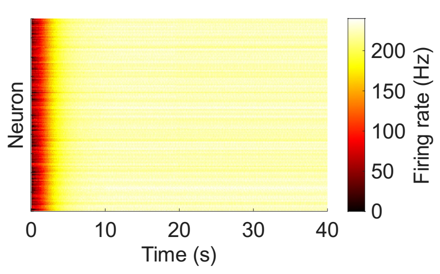

Figure 1g.

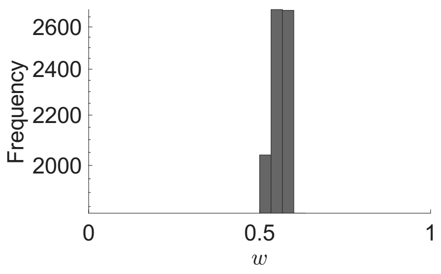

Figure 1h.

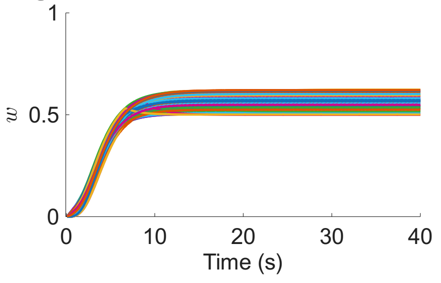

Figure 1i.

Figure 1. Typical network dynamics with additive or multiplicative STDP. **(A)** Firing rates of the network's neurons with additive STDP. **(B)** Weights, w, after learning with additive STDP. **(C)** Example of 100 synapses' weights with additive STDP. **(D-F)** Same as A-C, but with multiplicative STDP with Eq. S2 **(G-I)** Same as A-C, but with multiplicative STDP with Eq. S3

2 RESULTS

Figure 1 demonstrates stability and competition issues with typical additive STDP or multiplicative STDP implementations (Eqs. S1 and S2/S3 respectively) *without* resource dependence: Activity increases and weights converge to unimodal distributions. In the additive case, potentiation leads to increased activity, and increased activity leads to potentiation; this results in a unimodal distribution at the upper bound. In the multiplicative case, the distribution mean depends on the weight dependence: if weak, it is similar to additive, and if stronger, the weights converge to the weight dependence value. Under additive and multiplicative STDP *without* resource dependence, all neurons are recruited and all fire at the maximum firing rate allowed under the refractory constraint.

To explore whether a nonlinear STDP rule (Eq. S4) *with* resource dependence has an effect on the weight distribution as observed by Gütig et al. (2003) μ was systematically changed, Fig. 2: The weight distribution is similar regardless of the nonlinearity, suggesting that the weight distribution is shaped by the dependence on limited resources.

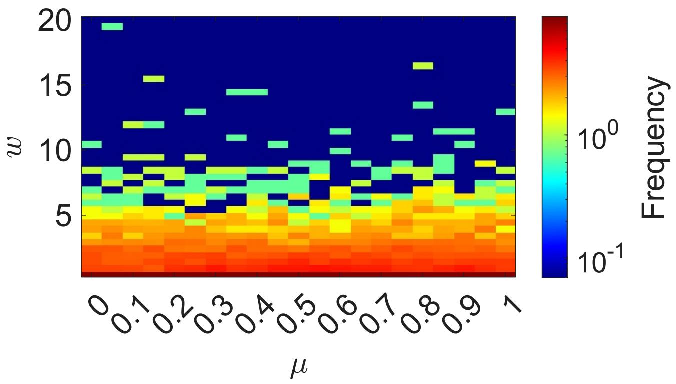

Figure 2. Recurrent weight frequency as a function of μ. The distribution of recurrent weights is unchanged.

REFERENCES

Gütig, R., Aharonov, R., Rotter, S., and Sompolinsky, H. (2003). Learning Input Correlations through Nonlinear Temporally Asymmetric Hebbian Plasticity. *The Journal of Neuroscience* 23, 3697–3714. doi:10.1523/jneurosci.23-09-03697.2003
